# Reirradiation for Rare Head and Neck Cancers: Orbit, Auditory Organ, and Salivary Glands

**DOI:** 10.7759/cureus.22727

**Published:** 2022-02-28

**Authors:** Hideya Yamazaki, Gen Suzuki, Norihiro Aibe, Hiroya Shiomi, Ryoong-Jin Oh, Ken Yoshida, Satoaki Nakamura, Mikio Ogita

**Affiliations:** 1 Radiology, Kyoto Prefectural University of Medicine, Kyoto, JPN; 2 Radiation Oncology, Miyakojima Image Guided Radiation Therapy (IGRT) Clinic, Osaka, JPN; 3 Radiology, Kansai Medical University, Hirakata, JPN; 4 Radiation Oncology, Kansai Medical University, Hirakata, JPN; 5 Radiotherapy, Fujimoto Hayasuzu Hospital, Miyakonojo, JPN

**Keywords:** recurrent, salivary gland, auditory organ, orbit, reirradiation

## Abstract

We analyzed the efficacy and toxicity following reirradiation for locoregional recurrence of rare head and neck tumors. We retrospectively analyzed 17 patients who had received reirradiation for rare head and neck tumors. Primary tumor sites included nine ears (auditory organ), four salivary glands, and four orbits. The median follow-up time was 13.2 months for surviving patients. The median survival time was 12.6 months with one- and two-year survival rates of 53.1% and 44.3%, respectively. Nine out of 17 patients experienced local failure. The one- and two-year local control rates were 42.4% and 31.8%, respectively. The median survival times were 12.6, 5.3, and 11.0 months for orbit, auditory organ, and salivary glands, respectively. Three patients experienced grade 3 toxicity, including meningitis, brain necrosis, and facial nerve disorders. No grade ≥4 toxicities were observed. Reirradiation of rare head and neck tumors is feasible, with acceptable toxicity.

## Introduction

Radiotherapy with or without systemic therapy is a standard treatment approach for locally advanced laryngeal and pharyngeal squamous cell carcinoma (SCC). Recent advancements in multidisciplinary approaches, both in radiotherapy and systemic therapy, have improved outcomes; however, locoregional recurrence is a predominant pattern of failure, which has been reported in up to 50% of cases [1-2]. For those patients, surgery is a curative salvage treatment to prolong survival; however, it is difficult in almost all cases and the majority received palliative chemotherapy with limited efficacy [3]. Thus, reirradiation could be an option for unresectable head and neck cancer to alleviate symptom palliation and improve oncologic outcomes [4-6]. Reirradiation is a challenging field for anticipating severe toxicities, including those that are lethal [4-7]. As many heterogeneous malignancies arise from many locations with various histological species, there are few reports of reirradiation for rare tumors, except for the oral and laryngopharyngeal areas. Therefore, we examined the outcome of reirradiation for rare tumors, including of the eye, ear, and salivary glands, based on multi-institutional data accumulation [8]. The purpose of the present study was to analyze the efficacy and toxicity of reirradiation for very rare tumors in the head and neck.

## Materials and methods

Patients with a recurrent very rare head or neck tumor treated at five Japanese institutes between 2002 and 2018 were recruited.

Patients with histologically confirmed recurrent and very rare diseases after the previous radiotherapy with significant overlap were included. We defined “rare disease” incidence < 0.06% = 600/1000000 as according to Rare Diseases Act of 2002 [9]. 

We used the following inclusion criteria: histology confirmed by pathology, reirradiation as radiotherapy performed after previous radiotherapy of 30 Gy/10 fractions (equivalent 2-Gy fractions = EQD2 ≥36 Gy, using α/β = 10 Gy) or more, pathological or radiological diagnoses obtained for patients presenting for salvage therapy after curative treatment including radiotherapy (definitive or postoperative), no distant metastasis, an Eastern Cooperative Oncology Group performance status of 0-2, and recurrence after curative treatment including surgery or chemotherapy with radiotherapy. Patients with distant metastasis or lymph node metastasis without local failure, and (c) palliative radiotherapy for symptomatic relief were excluded.

Fifteen patients underwent stereotactic radiotherapy (CyberKnife) and two underwent intensity-modulated radiotherapy (IMRT). Visible tumor on imaging studies was defined as gross tumor volume (GTV) and was expanded to the planning target volume (PTV) with an additional adequate margin. Patients were treated with a median dose of 30 Gy (range, 20-60 Gy) in a median of five fractions (range, 5-20 fractions). Normalized total doses in 2 Gy fraction = EQD2 were estimated according to the following equation: EQD2 = n×d×((α/β)+d)/((α/β)+2), where n is the number of treatment fractions, d is the dose per fraction in Gy, and α/β = 10 Gy.

Toxicity was evaluated according to the Common Terminology Criteria for Adverse Events Version 4.0.

This study was conducted in accordance with the Declaration of Helsinki and with the institutional review board (IRB, i.e., ERB-C-1330-3 in Kyoto Prefectural University of Medicine) permission from each institution.

Statistical analysis

All statistical analyses were performed using Stat-view 5.0 statistical software (SAS Institute, Cary, NC, USA) and R-stat [10]. A chi-square test was used in the analysis of frequencies. The student’s t-test for normally distributed data and the Mann-Whitney U-test for skewed data were used to analyze the comparison of means. Survival data and cumulative incidences were estimated using the Kaplan-Meier method and examined for significance using a log-rank test. Cutoff values were set at the median or average value of each variable unless otherwise stated. p < 0.05 was used as a value of statistical significance.

## Results

Local control rate and overall survival rate

Seventeen patients (median age, 69 years; range, 33-90 years) were collected from multi-institutional data. Patients were treated with a median dose of 30 Gy (range, 20-60 Gy) in a median of five fractions (range, 5-20 fractions). Table 1 shows patient characteristics. 

**Table 1:** Patient characteristics. PTNO, number of patients; EQD2Gy, n × d ([α/β] + d)/([α/β] + 2); n, number of treatment fractions; d, dose per fraction in Gy; α/β = 10 Gy; ACC, adenoid cystic carcinoma; BCC, basal cell carcinoma; SCC, squamous cell carcinoma; MFH, malignant fibrous histiocytoma

Variables	Group	Median or PT NO	(%) or range
Age		69	33-90
Gender	Female	12	(66.7%)
	Male	5	(27.8%)
Primary site	Eye	4	(22.2%)
	Ear	9	(50.0%)
	Salivary gland	4	(22.2%)
Disease site	Primary only	15	(83.3%)
	Primary + Lymph node	2	(11.1%)
Histology	ACC	1	(5.6%)
	Acinic cell carcinoma	1	(5.6%)
	Adenocarcinoma	4	(22.2%)
	BCC	2	(11.1%)
	MFH	1	(5.6%)
	SCC	8	(44.4%)
Chemotherapy	No	11	(61.1%)
	Yes	6	(33.3%)
Previous surgery	No	8	(44.4%)
	Yes	9	(50.0%)
Gross tumor volume	cm^3^	18	1-128
Prescribed dose	(Gy)	30	20-60
Fractionation	(fractions)	5	5-20
EQD2Gy	(Gy)	34.7	23.3-65
Interval between treatment	(months)	15.2	3-207
Previous prescribed doses	(Gy)	60	35-81.4
Previous fractionation	(fractions)	30	5-41
Previous EQD2	(Gy)	60	36.5-82.7

**Figure 1:**
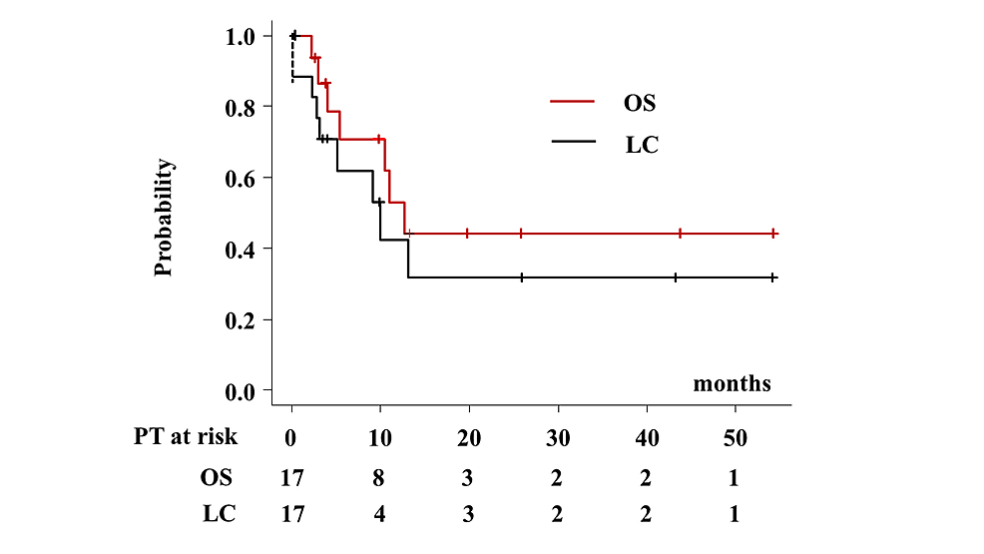
Local control rate and overall survival rate.

Primary tumor sites included nine ears (auditory organs), four salivary glands, and four eyes (orbits). With a median follow-up period of 9.8 months (range 0.37-54.1 months), and 13.2 months for surviving patients, the median survival time was 12.6 months with one- and two-year survival rates of 53.1% [95% confidential interval (95% CI) = 23.3%-76.0%] and 44.3% (95% CI = 16.8%-68.9%), respectively (Figure 1a).

Among the 17 patients, nine experienced local failure during the follow-up period. The one- and two-year local control rates were 42.4% (95% CI = 15.4%-67.4%) and 31.8% (95% CI = 0.85%-58.6%), respectively (Figure 1). The detailed treatment schedule and outcomes are shown in Table 2.

**Table 2:** Analysis for local control and survival rate. CI, confidence interval; LC, local control; OS, overall survival; SCC, squamous cell carcinoma; GTV, gross tumor volume

Variable	Strata	LC rate		OS rate	
		2-year LC	p-value	2-year OS	p-value
Age (years)	≤ 70 (n=9)	22.2%	0.0719	28.9%	0.165
	≥ 71 (n=8)	53.3%		60.0%	
Gender	Female	33.3%	0.883	40.9%	0.879
	Male	40.0%		50.0%	
Location	Eye and salivary glands	46.7%	0.283	50.0%	0.202
	Ear	27.8%		35.0%	
Histology	Other	35.6%	0.822	58.3%	0.152
	SCC	37.5%		20.8%	
Previous surgery	NO	37.5%	0.755	37.5%	0.308
	Yes	44.4%		50.0%	
Chemotherapy	NO	34.1%	0.574	53.0%	0.436
	Yes	50.0%		25.0%	
GTV	≤ 25 cm^3^	71.4%	0.347	43.8%	0.413
	> 25 cm^3^	18.7%		41.7%	
Interval between treatment	≤ 12 months	38.9%	0.536	27.3%	0.168
	> 12 months	31.2%		57.1%	
Prescribed doses	EQD2 ≤ 40 Gy	47.4%	0.205	51.4%	0.456
	EQD2 > 40 Gy	25.0%		36.5%	

**Figure 2:**
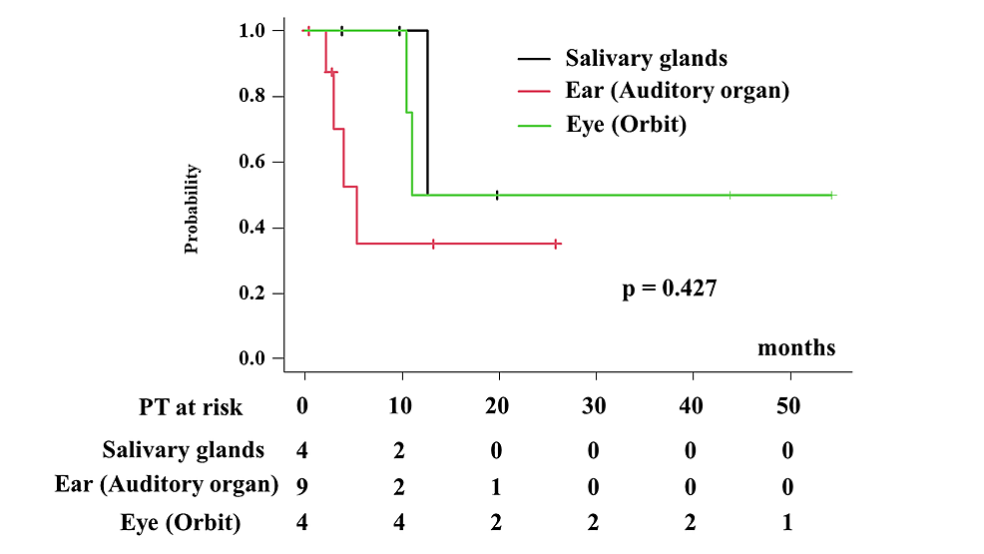
Overall survival rate according to primary sites.

The results of the analysis for the overall survival rate and local control are shown in Table 2. The median survival times were 12.6, 5.3, and 11.0 months for eye (orbit), ear (auditory organ), and salivary glands (p = 0.427, Figure 2), respectively. The one- and two-year overall survival rates were 100%, 35.0% (95% CI = 4.9%-96.9%), 50.0% (95% CI = 5.78%-84.5%), 50% (95% CI = 0.5%-91.0%), 35% (95% CI = 4.9%-96.9%), and 50% (95% CI = 5.78%-84.5%) (p = 0.427) for the eye (orbit), ear (auditory organ), and salivary glands, respectively (Figure 1b).

**Figure 3:**
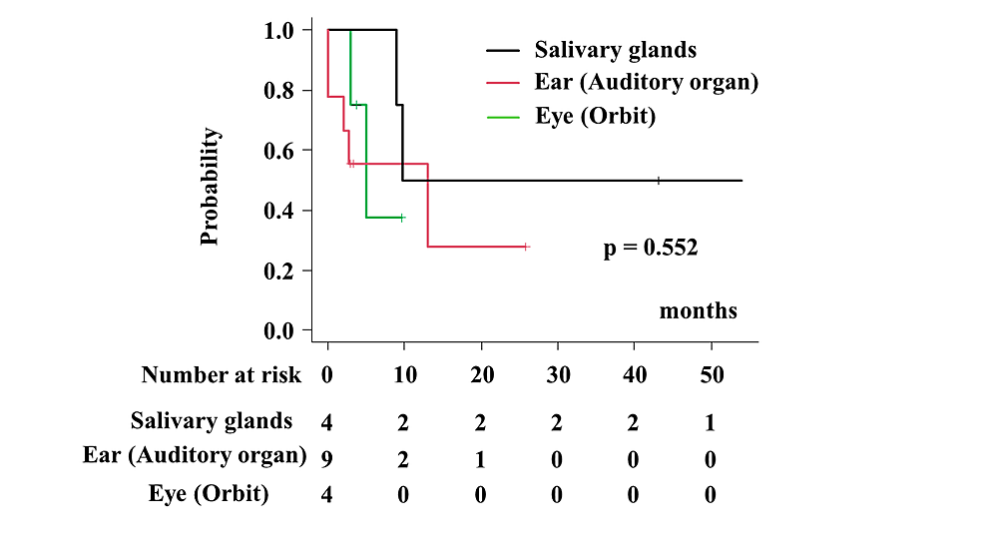
Local control rate according to primary sites.

The one- and two-year local control rates were 37.5% (95% CI = 1.1%-80.8%) at 9.7 months, 55.6% (95% CI = 20.4%-80.5%), 50.0% (95% CI = 5.78%-84.5%), and not available, 27.8% (95% CI = 1.5%-67.3%) and 50% (95% CI = 5.78%-84.5%) (p = 0.552) for the eye (orbit), ear (auditory organ), and salivary glands, respectively (Figure 3).

We did not find any statistically significant predisposing factors for local control or survival. We did not find any statistically significant predisposing factor for local control or overall survival rate (Table 3).

 Salivary gland carcinomas

**Table 3:** Detailed patients characteristics, treatment, and outcome of salivary gland tumor. SCC, squamous cell carcinoma; AD, adenocarcinoma; MFH, malignant fibrous histiocytoma; BCC, basal cell carcinoma; CTX, chemotherapy; fr, fraction; ReRT, reirradiation; LC, local control; NED, no evidence of disease; DT, death for local tumor; AWD, alive with disease; EQD2Gy = n × d ([α/β] + d)/([α/β] + 2); n, number of treatment fractions; d, dose per fraction in Gy, α/β = 10 Gy.

PTNO	Gender	Age	Location	Histology	Previous surgery	CTX	Initial RT schedule	Initial EQD2 (Gy)	Interval (months)	ReRT schedule	ReRT EQD2 (Gy)	GTV (cm^3^)	LC	LC (months)	Status	OS (months)	Toxicity grade 3
1	F	73	Parotid	AD	Yes	No	37 Gy /5 fr	53.7	4.7	20 Gy / 5 fr	23.3	20.1	Yes	43.1	AWD	43.7	No
2	M	76	Parotid	ACC	No	No	68 Gy /41 fr	66.1	15.3	27 Gy / 5 fr	34.7	25.8	Yes	54	NED	54.2	No
3	M	72	Parotid/cavernous sinus invasion	AD	Yes	No	60 Gy /30 fr	60	22.2	27 Gy / 5 fr	34.7	10.7	No	9	DT	10.5	Brain necrosis (lateral lobe)
4	F	75	Parotid	SCC	No	No	37 Gy /8 fr	45.1	3.3	20 Gy / 5 fr	23.3	16.3	No	9.8	DT	11.0	No

Four patients showed parotid gland malignancies which included two adenocarcinomas, one acinic cell carcinoma, and one SCC. Table 3 showed a detailed treatment schedule and outcome. One patient with SCC experienced local recurrence at 9.8 months after 20 Gy/five fraction of reirradiation and resulted in death 11 months later. On the other hand, one patient with acinic cell carcinoma obtained a complete response with 27 Gy/five fraction and was alive without any evidence of disease at 54 months after reirradiation.

Auditory organ cancer

Nine patients showed auditory organ malignancies which included eight external auditory canals (six scc, one adenocarcinoma, and one basal cell carcinoma), and one auricle basal cell carcinoma. Table 4 showed a detailed treatment schedule and outcome. Six patients with SCC showed a 25% of one-year survival rate, whereas the other three patients showed a 66.7% of one-year survival rate (p = 0.515).

**Table 4:** Detailed patients characteristics, treatment, and outcome for tumor of auditory organ. SCC, squamous cell carcinoma; AD, adenocarcinoma; MFH, malignant fibrous histiocytoma; BCC, basal cell carcinoma; CTX, chemotherapy; ia = intra arterial infusion chemotherapy, CTX = chemotherapy, fr, fraction; ReRT, reirradiation; LC, local control; NED, no evidence of disease; DT, death for local tumor; AWD, alive with disease; DTNM, death for locoregional and distant failure; GTV, gross tumor volume *GTV included not only primary recurrence but also lymph node metastasis EQD2Gy = n × d ([α/β] + d)/([α/β] + 2); n = number of treatment fractions; d = dose per fraction in Gy, α/β = 10 Gy.

PTNO	Gender	Age	Location	Histology	Previous surgery	CTX	Initial RT schedule	Initial EQD2 (Gy)	Interval (months)	ReRT schedule	ReRT EQD2 (Gy)	GTV (cm^3^)	Local control	LC (months)	Status	OS (months)	Toxicity grade 3
1	M	67	Auricle	BCC	Yes	No	80 Gy /35 fr	81.9	15.6	30 Gy / 5 fr	40.0	1.4	No	0	AWD	2.7	No
2	F	50	External auditory canal	BCC	No	No	60 Gy /30 fr	60	13.8	54 Gy/ 15 fr	61.2	12.8	No	13	AWD	13.2	No
3	F	33	External auditory canal/skull base	AD	No	No	38.25 Gy /17 fr	39	7.3	32 Gy / 5 fr	43.7	55.4*	No	2.1	DTN	2.2	No
4	F	52	External auditory canal	SCC	Yes	Yes	80 Gy /40 fr	80	6.8	24 Gy / 8 fr	26.0	24.5	No	2.7	AWD	2.7	No
5	F	79	External auditory canal	SCC	Yes	Yes	35 Gy /14 fr	36.5	2.3	24 Gy / 8 fr	26.0	25.3	Yes	3	AWD	0.4	No
6	F	64	External auditory canal	SCC	No	No	60 Gy /30 fr	60	8.5	30 Gy / 8 fr	34.4	42.5	Yes	3.3	DTNM	4.0	No
7	F	66	External auditory canal	SCC	No	Yes	70.4 Gy /27 fr	74	22.4	30 Gy / 5 fr	40.0	43.7	Yes	25.8	AWD	25.8	No
8	F	64	External auditory canal	SCC	No	Yes	70 Gy /35 fr	70	21.1	36 Gy / 6 fr	48.0	22.9	No	0	DT	5.4	Meningitis (s/o tumor involvement)
9	M	58	External auditory canal	SCC	No	Yes (ia)	60 Gy /30 fr	60	6.2	60 Gy/ 20 fr	65.0	128*	Yes	3	DTNM	3.0	Facial nerve palsy

Orbital cancer

Four patients showed orbital malignancies which included one adenoid cystic carcinoma (ACC), one apocrine adenocarcinoma, one malignant fibrous histiocytoma, one ACC, and squamous cell carcinoma (SCC) each. Detailed treatment schedules and outcomes were shown in Table 5. One patient with orbital squamous cell cancer treated with 35 Gy/seven fractions of reirradiation recurred 3.8 months after reirradiation.

**Table 5:** Detailed patients characteristics, treatment, and outcome for tumor of eye. SCC, squamous cell carcinoma; MFH, malignant fibrous histiocytoma; ACC, adenoid cystic carcinoma; CTX, chemotherapy; fr, fraction; ReRT, reirradiation; AWD, alive with disease; DTM, death for local and distant failure; LC, local control; EQD2Gy = n × d ([α/β] + d)/([α/β] + 2); n, number of treatment fractions; d, dose per fraction in Gy, α/β = 10 Gy.

PTNO	Gender	Age	Location	Histology	Previous surgery	CTX	Initial RT schedule	Initial EQD2 (Gy)	Interval (months)	ReRT schedule	ReRT EQD2 (Gy)	GTV (cm^3^)	LC	LC (months)	Status	OS (months)	Toxicity grade 3
1	F	39	Orbit	ACC	Yes	Yes	66 Gy /33 fr	66	22.8	30 Gy / 5 fr	40.0	1.0	No	3.0	DTM (Lung meta)	12.7	No
2	M	84	Orbit	Apocrine adenocarcinoma	Yes	No	81.4 Gy / 37 fr	82.8	207.2	25 Gy/ 5 fr	31.3	16.0	Yes	5.0	AWD	19.8	No
3	F	90	Eyelids	MFH	Yes	No	37 Gy /5 fr	53.7	4	20 Gy/ 5 fr	23.3	17.7	Yes	9.7	AWD	9.8	No
4	F	79	Orbit	SCC	Yes	No	60 Gy /30 fr	60	10.6	35 Gy/ 7 fr	43.8	7.4	No	3.8	AWD	3.8	No

Toxicity

Three patients experienced grade 3 toxicity, including meningitis, brain necrosis, and facial nerve disorders (Table 2). No grade ≥4 toxicities were observed.

## Discussion

Herein we present the efficacy and toxicity of reirradiation for rare head and neck cancers using multi-institution accumulated data [8]. This is the largest series of reirradiation cohorts for those rare diseases, to the best of our knowledge. In general, with the advent of radiotherapy techniques, reirradiation has gained attention in cases of unresectable recurrent head and neck cancers, mainly for oral, laryngeal, and pharyngeal SCC. For those major diseases, Strojan et al. reviewed and reported that the two-year overall survival rate was generally 10%-30%; grade 3-4 late effects were common in 40%, and grade 5 due to carotid rupture, hemorrhage, sepsis, etc. were found in approximately 10% [4]. Lee et al. conducted a meta-analysis of IMRT/stereotactic radiation therapy (SRT) and found a two-year survival rate of 30%-46%; with toxicities of grade ≥3 was 9.6%-26% [5-6]. Particle therapy is now in use in the clinic [11-13], and the National Health Insurance System in Japan has covered particle beam therapy for head and neck malignancies, except for oral, laryngeal, and pharyngeal SCC, since April 2018. Therefore, particle therapy mainly treats malignancies, except for oral, laryngeal, and pharyngeal SCC [11-13]. Furthermore, boron neutron capture therapy (BNCT) has been covered by the National Health Insurance since 2021 for recurrent/locally advanced malignancies of head and neck cancer and has shown good preliminary outcomes even after reirradiation [14]. We hope that these advanced techniques will improve the outcome after reirradiation in the near future.

Salivary gland carcinomas

Salivary gland carcinomas are very rare malignant epithelial tumors. The last World Health Organization classification (2017) counts more than 20 malignant histologic types [15]. Salivary gland malignancies account for 0.5%-1.2% of all cancers and 5% of head and neck cancers (incidence; 11.95/1,000,000 person-years) [15-16]. The parotid gland is a major origin of malignant tumor, followed by the submandibular, sublingual, and minor salivary glands. The most common malignant tumors are mucoepidermoid carcinoma, ACC, carcinoma ex-pleomorphic adenoma, adenocarcinoma, and SCC [15]. Vischioni et al. reported that a carbon ion radiotherapy is a good option for retreatment of inoperable recurrent salivary gland tumors, and GTV of retreated tumors might significantly influence local control [12]. More than 80% of acinic cell carcinoma originated from the parotid gland, followed by submandibular glands (4%), and intraoral minor salivary glands (17%) [15]. In our series, acinic cell carcinoma showed a good response to reirradiation and remained controlled 54 months later, which concurs with a previous report [17]. Primary SCC is usually found in the parotid gland [14]. Although there is evidence implicating high-risk human papilloma virus (HPV), the etiology of malignant transformation is unknown [15]. Evidence from the literature suggests that the true incidence of SCC may be approximately 0.75%-1% [15-16]. Zhan et al. found that 10% of all parotid cancers (n = 3,155) were parotid adenocarcinomas not otherwise specified in the surveillance, epidemiology, and end results (SEER) database [18]. Patients with stage III-IV disease who underwent surgery and radiotherapy had better overall survival than those who underwent surgery alone (51% vs. 41%; p < 0.001) [18]. Parotid adenocarcinoma is an aggressive disease with frequent regional metastasis and low survival rates. In our series, one patient showed intra-skull involvement (cavernous sinus involvement) and underwent reirradiation; however, it resulted in tumor death.

 Auditory organ cancer

Neoplasms of the auditory organ are a rare disease with an annual incidence of (1-6/1000000) for SCC [19-20]. Surgery is the major initial therapy for patients with cancer of the external auditory canal, however, it is controversial because expert techniques are needed for rarity and the complex anatomy of the temporal bone including cranial nerves, internal carotid artery, and brain. Takenaka et al. made a meta-analysis of 29 articles published between 2006 and 2013 [21]. The two-year overall survival rates for recurrent/locally advanced SCC and non-SCC were 58% and 100%, respectively. There are a few reirradiation series for external auditory canal malignancies [13, 22]. Our data also implied poorer outcome of SCC than non-SCC. Matsuo et al. reported further second reirradiation (third radiotherapy) performed by BNCT even after reirradiation using particle therapy [23]. 

Orbital cancer

The incidence of adenocarcinoma originating from the apocrine gland is 0.049-0.173 per 1000000 [24]. Bonavolontà et al. analyzed 2,480 orbital space-occupying lesions in their retrospective review and found that non-Hodgkin lymphoma was the most common malignant neoplasm (12%) [25]. Lacrimal gland lesions were benign in 64% (154/241) of cases, whereas the most common malignant tumor was ACC (18%) [25]. Local control does not necessarily prevent delayed distant metastases in the future for ACC of the lacrimal gland [26]. Our case was in line with this report and showed lung metastasis and the death of the tumor. In addition to other head-neck lesions, particle therapy has been used to treat ACC [11-14, 26-27]. Gordon et al. reported a clinical case of recurrent orbital hemangiopericytoma that was irradiated five times [28]. Apocrine adenocarcinoma is a cutaneous adnexal malignancy with a slow-growing but refractory nature, which rarely arises from ocular structures, and sometimes initially present with an orbital tumor [29]. Our case also initially showed an orbital tumor with invasion to the paranasal sinus. The patient received proton beam radiotherapy, although there was recurrence at the lymph node and a peripheral lesion three years later, for which she underwent salvage surgery. She further developed local recurrence at the nasal cavity and maxillary sinus nine years later and underwent salvage surgery with positive margins. Subsequently, recurrence occurred yet again, and salvage surgery and reirradiation were performed. She is alive with a stable tumor 19.8 months after reirradiation. Differential diagnosis of periocular malignant fibrous histiocytoma is difficult from leiomyosarcoma, atypical fibroxanthoma, sarcomatoid carcinoma, and atypical fibroxanthoma [29]. The mainstay of treatment is complete surgical excision with wide margins, and histologic margin control should be considered in addition to adjuvant radiotherapy. Our patient also showed postoperative recurrence; initial radiotherapy could not control the tumor, although additional irradiation was able to inhibit sustain tumor growth. The SCC of the orbit is very rare and has been cited in reports describing the treatment outcome of BCC [25].

This study had some limitations. The retrospective nature of this study included small sample size and limited follow-up time, which may limit its application. Heterogeneous tumor location and histology prevented concrete conclusions and may have selection bias. However, our study is one of the few studies on reirradiation for very rare head and neck diseases and provides valuable information. At last, the lack of detailed information on the previous radiotherapy is a limitation of this study. As we could not reach detailed dose-volume information about organs at risk for other institutions, we added policy in our institution. We made a summation of dose distribution [previous radiotherapy (via DICOM if it was performed in other institutions) and reirradiation] using the image fusion technique. We assumed that organs at risk (near target volume) could receive full-field exposure to the prescription dose if previous plans are unavailable. As we understood that normal tissue recovery could exist after prior radiotherapy, we applied dose constrain including normal tissue recovery ratio depending on each tissue (i.e., 50% dose reduction of the previous dose could be done in the spinal cord by recovery after one to three years interval) proposed by experienced institutions although not validated [30]. We used dose constrain using composite dose distribution (previous radiotherapy plus reirradiation); optic chiasm [dose limit Max to 0.1cc (EQD2) = 54 Gy in α/β = 2.5 Gy], retina (50 Gy), spinal cord (50 Gy), and brainstem (64 Gy) [30]. 

## Conclusions

Head and neck malignancies can be treated with reirradiation but anticipating severe toxicity. This report provides evidence that reirradiation can be an effective treatment option for recurrent rare tumors. Reirradiation can be served as a potential option for patients who are not candidates or decline surgical intervention or systemic therapy. Reirradiation for extremely rare head and neck cancers: orbit, auditory organ, and salivary glands is feasible, with acceptable toxicity.
